# Mineral Intake and Status of Cow's Milk Allergic Infants Consuming an Amino Acid-based Formula

**DOI:** 10.1097/MPG.0000000000001655

**Published:** 2017-08-22

**Authors:** Bryan M. Harvey, Simone R.B.M. Eussen, Lucien F. Harthoorn, A. Wesley Burks

**Affiliations:** ∗ Children's Investigational Research Program, LLC (ChiRP), Bentonville, AR; † Nutricia Research, Nutricia Advanced Medical Nutrition, Utrecht, The Netherlands; ‡ University of North Carolina, School of Medicine, Chapel Hill, NC.

**Keywords:** amino acid-based formula, calcium, cow's milk allergy, iron, mineral, phosphorus

## Abstract

Data on the mineral status of infants with cow's milk allergy (CMA) consuming an amino acid-based formula (AAF) have not been published. The present study aims to assess mineral status of term infants age 0 to 8 months diagnosed with CMA receiving an AAF for 16 weeks. Serum concentrations of calcium, phosphorus, chloride, sodium, potassium, magnesium, and ferritin were determined in 82 subjects at baseline and in 66 subjects after 16 weeks on AAF using standard methods and evaluated against age-specific reference ranges. In addition to this, individual estimated energy and mineral intakes were compared to Adequate Intakes defined by the European Food Safety Authority and the US Institute of Medicine. The results of this study show that the AAF was effective in providing an adequate mineral status in infants with CMA. The vast majority of infants aged 0 to 6 months (formula only) and aged 6 to 12 months (formula and complementary foods) had adequate mineral intakes.

**What Is Known**

- Amino acid-based formulae are well-tolerated, effective, and safe, and effectively promote growth in infants with cow's milk allergy.

**What Is New**

- The present study shows that an amino acid-based formula is effective in providing adequate dietary mineral intake and maintaining mineral status in infants with cow's milk allergy.
- These data add to the understanding of the nutritional status of infants with cow's milk allergy consuming an amino acid-based formula.

Cow's milk allergy (CMA) is the most common food allergy in infancy, affecting up to 5% of infants in their first year of life (1). The symptoms of food allergy are gastrointestinal, cutaneous, and respiratory (1). Fundamental to CMA management is complete elimination of offending proteins. For infants who are breast-fed, this requires mothers eliminating all cow's milk protein from their diet. If breast-feeding is not possible, a suitable cow's milk-free formula is required. Hypoallergenic formulae such as extensively hydrolyzed formula (eHF) or amino acid-based formula (AAF) should be the formula of choice (2). An eHF is recommended for infants with mild to moderate CMA, whereas initiation with an AAF is recommended for more severe or complex allergy or when symptom-resolution with eHF fails (2). Dietary management of CMA with an AAF has been proven to be well-tolerated, effective, and safe; adequate infant growth on an AAF has widely been reported (3–5). Data on mineral status of infants consuming AAF have, to our knowledge, not, however, been published. Yet, eliminating dairy products in diets of CMA infants, as well as coexisting feeding problems, can result in removing a substantial source of minerals, including calcium and phosphorus, which are involved in essential body functions (6). The aim of this study was to assess mineral status (calcium, phosphorus, chloride, sodium, potassium, magnesium, and iron) of infants receiving an AAF for 16 weeks. In addition to this, dietary mineral intakes were estimated and evaluated against Adequate Intakes (AIs).

## METHODS

In a prospective, randomized, double-blind controlled study, full-term infants age 0 to 8 months with confirmed Immunoglobulin E (IgE) or non-IgE-mediated CMA were randomized to receive an AAF (n = 110) with or without synbiotics (short-chain and long-chain oligosaccharides [1.0 g/100 kcal], *Bifidobacterium breve* M-16 V [2.16 × 10^9^ CFU/100 kcal]) for 16 weeks (3,4). Details about the study formulae (Neocate; SHS International Ltd., Nutricia Advanced Medical Nutrition, Liverpool, UK) can be found in Supplemental Digital Content, Table 1.

CMA was diagnosed according to specified criteria listed in Supplemental Digital Content, Table 2. Infants could have received dietary treatment with an alternative commercially available hypoallergenic formula before study entry. Solid foods free of milk and other allergenic proteins dependent on documented allergies were part of the subject's diet as advised by their physician. Subjects were recruited between April 2008 and March 2012 within 29 participating sites in the United SA.

Primary outcomes were growth and formula tolerance and have been reported previously (3,4). The clinical trial was registered as NCT00664768 and medical ethical approval was obtained by the Chesapeake Institutional Review Board (Columbia, MD). All parents/guardians gave written informed consent for their children to participate.

Mineral status was assessed by standard analyses of blood samples obtained at baseline and at 16 weeks, and included calcium, phosphorus, chloride, sodium, potassium, magnesium, and iron (ferritin). Furthermore, hemoglobin, albumin, and total protein were determined. These laboratory measurements are routinely used to assess the nutritional status of our target group (7). All blood samples were analyzed in 1 central laboratory (Covance Central, Laboratories Services Inc, Indianapolis, IN). Differences in blood chemistry parameters (at baseline, week 16 and change from baseline) between the study products have been analyzed before and were not found to be statistically significant or clinically relevant (3). Therefore, blood chemistry parameters are presented for the overall population.

Average daily formula intake consumed by each subject was recorded in 3-day food diaries at weeks 2, 4, 8, and 16 of the study. From these intake data, individual energy and mineral intakes were calculated based on study product composition. Energy intakes were evaluated against Dietary Reference Values for energy of the UK Scientific Advisory Committee on Nutrition (8) and the US Institute of Medicine (IOM) (9). Due to the correlation between energy requirements and energy intake, it was not possible to estimate the prevalence of inadequate energy intake without information on individual energy requirements (9,10). Mineral intakes (calcium, phosphorus, chloride, sodium, potassium, magnesium, and iron) were evaluated against AIs defined by the European Food Safety Authority (EFSA) (11) and the IOM (12,13). The AI is the daily mean nutrient intake when healthy, full-term infants are consuming human milk, but cannot be used to estimate the proportion of infants with inadequate intakes (12). Therefore, intake was evaluated qualitatively. If median intake was above the AI, the prevalence of inadequate intake was stated as “low”. When this was not the case, the adequacy of the diet could not be evaluated (“no statement”) (10). To estimate total mineral intake, that is, intake from both formula and complementary foods, we used published intake data made by complementary foods (ie, all foods other than infant formula/human milk/cow's milk) from the UK Diet and Nutrition Survey of Infants and Young Children (14). In this survey, no estimates are given of the contribution of complementary foods to intakes of phosphorus, chloride, potassium, and magnesium, and therefore only intake data from formula are presented for these minerals.

## RESULTS

Baseline characteristics of the enrolled subjects are presented in Supplemental Digital Content, Table 3. Average age of infants at inclusion was 4.6 ± 2.5 months (mean ± SD). Out of the 110 infants included in the study, 82 (75%) had blood parameters analyzed at baseline and 66 (60%) after 16 weeks on AAF. Reasons for dropout are presented in Supplemental Digital Content 4, Figure 1.

At baseline and after 16 weeks on AAF, mean blood concentrations of all minerals were within the specified reference ranges set for the corresponding ages of the infants (Table 1). Among some minerals, there was a number of individual values at baseline that were below the reference ranges for age, that is, calcium (n = 1), phosphorus (n = 1), chloride (n = 1), sodium (n = 1), and ferritin (n = 6), whereas at week 16 only ferritin concentrations remained below the reference range for age for a number of individuals (n = 15) (Table 1). Mean values of hemoglobin, albumin and total protein were within reference ranges for age at baseline and after 16 weeks (Table 1).

The median daily intake of energy ranged between 467 and 588 kcal/day in boys and between 421 and 515 kcal/day in girls younger than 6 months of age, that is, below the age-group specific estimated energy requirement (EER) (Supplemental Digital Content 5, Table 4). For infants older than 6 months, the gap between energy intake from formula and EER was bigger. This was especially true for boys age 10 and 12 months who met approximately 60% of their EER from formula (vs 94% at 0–3 months), compared with 80% in girls of the same age (vs 85% at 0–3 months).

The median intakes of the minerals for subjects below 6 months are presented in Table 2. Full intake distributions for all ages are provided in Supplemental Digital Content 6, Table 5. For all minerals, median intakes of infants age 0 to 6 months was above the AI, indicating a low prevalence of inadequate intakes (Table 2). For calcium and phosphorus (Supplemental Digital Content 6, Table 5A and B), median intakes from formula in both boys and girls age 7 to 12 months was above the AI indicating a low prevalence of inadequate intakes. For iron (Supplemental Digital Content 6, Table 5C), median intakes from formula alone in boys and girls age 7 to 12 months was above or close to the AI defined by the EFSA (8 mg/day) and median intakes from formula and complementary foods was above or close to the AI defined by the IOM (11 mg/day). Overall, suggesting a low prevalence of inadequate iron intake.

Median sodium intakes from formula alone ranged between 164 and 209 mg/day in 7 to 12-month-old boys and between 187 and 254 mg/day in 7 and 12-month-old girls. Taking intake from complementary foods into account resulted in an increase of the median intake to the range of 424 to 614 mg/day for boys and 432 to 659 mg/day for girls (Supplemental Digital Content 6, Table 5E), that is, a level above the AI of 170 to 370 mg/day, indicating a low prevalence of inadequate sodium intake. For chloride, potassium and magnesium (Supplemental Digital Content 6, Table 5D to G), median intakes of formula alone in 7 to 12-month-olds were below the AI and therefore no statement about the prevalence of inadequate intakes could be made. Due to the absence of data in the UK Diet and Nutrition Survey, intake from complementary foods could not be considered for these minerals.

## DISCUSSION

The present study shows that AAF with or without synbiotics, which have been reported previously to be equally tolerated and support normal growth (3,4), as part of an age-adapted diet, are effective in providing an adequate mineral intake and status in CMA infants.

The vast majority of infants age 0 to 6 months (formula only) and age 7 to 12 months (formula and complementary foods) had mineral intakes above the AI, indicating a high likelihood of achieving adequate intakes. For those infants with a mineral intake below the AI, this was related to a lower formula volume intake compared with what would have been expected based on the infant's EER (data not shown). Intake levels of calcium and phosphorus from formula alone were far above AIs set by the EFSA and IOM, especially in the first half year of infants’ life. The AAF used in this study is suitable for the whole first year of life, thus including the weaning period. The level of calcium and phosphorus in the AAF should therefore compensate for the low intakes of these nutrients in the merely dairy-free complementary diet of CMA infants. Moreover, compared with other countries (15) or Institutes (16), EFSA, and IOM set relatively low AIs for calcium and phosphorus. Neither the IOM (12,13) nor the EU Scientific Committee on Food (17) set a Tolerable Upper Intake Level (UL) of calcium and phosphorus for infants, but the P95 intake levels of both minerals remain far below the ULs of calcium and phosphorus for children age 1 to 3 years. Calcium and phosphorus levels in the study formulae comply with all compositional standards for infant formula applicable in the USA, EU, China, and Brazil.

Mineral status was within reference ranges for the vast majority of infants. At baseline, a few infants had individual blood mineral concentrations below reference ranges, whereas at week 16 all mineral concentrations, with the exception of ferritin, were within the age-specific range. Serum ferritin concentrations decreased during the study, a phenomenon also seen in healthy infants as they grow older (18). Prevalence of depleted iron stores (serum ferritin <12 μg/L (19)) found after 16 weeks of study, that is, 22%, are similar to those found in healthy infants (20). Other indices for assessing iron status, such as hematocrit, mean corpuscular volume, and total iron binding capacity, were all within reference ranges for age (data not shown) (3). Serum phosphorus concentrations also dropped slightly over the time of the study, in line with documented declines in phosphorus concentrations from 6 to 12 months of age (21), but remained above age-specific reference ranges.

This study has some limitations. First, the high number of subjects (40%) not available for analysis of the blood parameters. Baseline characteristics of the dropouts were comparable to the subjects included in the final analysis and the dropout rate was equal in both study arms. Therefore, it is not likely that this has influenced our findings. Secondly, in our study, some infants were already receiving an AAF at enrolment, and thus, baseline blood values cannot be strictly considered as “before intervention” values. Nevertheless, according to the study aim, the present analyses provide evidence that after 16 weeks of management with an AAF, mineral status of all participating infants was adequate. Finally, the use of the UK Diet and Nutrition Survey (14) has a limitation since it only presents data for a selected number of minerals. Moreover, estimates of nutrient intakes from complementary foods from this survey including healthy infants may be an overestimate for CMA infants given that food intakes overall may be poorer and consequences of excluding milk from the diet affects consumption of other food groups, such as breakfast cereals. This survey data have been used as a reasonable approximation in the absence of data on intakes of complementary foods by CMA infants.

In conclusion, these data further contribute to the knowledge about nutritional status and disease management of CMA infants by showing that an AAF with or without synbiotics is effective in providing an adequate mineral status in these infants. To assure normal-for-age mineral status by all CMA infants, it is necessary to provide them with regular medical and nutritional care to ensure adequate formula intakes and, if needed, additional supplementation.

## Supplementary Material

Supplemental Digital Content

## Supplementary Material

Supplemental Digital Content

## Supplementary Material

Supplemental Digital Content

## Supplementary Material

Supplemental Digital Content

## Supplementary Material

Supplemental Digital Content

## Supplementary Material

Supplemental Digital Content

## Figures and Tables

**TABLE 1:** Blood chemistry parameters at baseline (n = 82) and after 16 weeks on amino acid-based formula (n = 66)

		Baseline		After 16 weeks on AAF	
Parameter	Reference value	Mean (SD)	n (%)	Mean (SD)	n (%)
Ca, mmol/L	2.25–2.74	2.67 (0.14)	1 (1%)	2.62 (0.11)	0
P, mmol/L	1.36–2.62 (<1 y)	2.05 (0.25)	1 (1%)	1.97 (0.20)	0
	1.03–1.97 (≥1 y)	—		1.86 (0.24)	0
Cl, mmol/L	94–112	104 (3.2)	1 (1%)	104 (2.3)	0
Na, mmol/L	132–147	140 (3.3)	1 (1%)	140 (2.3)	0
K, mmol/L	3.7–5.6 (<1 y)	5.0 (0.52)	0	4.6 (0.29)	0
	3.4–5.4 (≥1 y)	—		4.6 (0.48)	0
Mg, mmol/L	0.70–0.98 (<30 day)	0.89 (0.05)	0	0.92 (0.05)	0
	0.66–1.03 (M; ≥30 day)	0.95 (0.06)	0	0.95 (0.07)	0
	0.78–0.98 (F; ≥30 day)	0.95 (0.05)	0	0.96 (0.07)	0
Ferritin, μg/L	≥12	61 (55)	6 (7%)	24 (18)	15 (22%)
Hemoglobin, g/L	100–200 (<30 day)	168 (4.6)	0	—	—
	100–140 (<5 mo)	115 (11)	4 (7%)	122 (8.5)	0
	105–135 (≥5 mo)	119 (9.4)	2 (8%)	121 (9.0)	2 (4%)
Albumin, g/L	24–48 (<30 day)	38 (2.3)	0	—	
	28–48 (≥30 day)	39 (5.2)	3 (4%)	41 (3.7)	0
Total protein, g/L	54–74 (<3 mo)	58 (3.4)	1 (4%)		
	55–71 (≥3 mo)	61 (6.5)	5 (9%)	64 (4.3)	0

Mean (SD) and number (%) of infants having a mineral status below the lowest range of the reference value. AAF = amino acid-based formula; Ca = calcium; Cl = chloride; K = potassium; Mg = magnesium; Na = sodium; P = phosphorus; y = years.

**TABLE 2:** Median (p50) intakes of minerals from formula alone for 0 to 3-month-old boys, 4 to 6-month-old boys, 0 to 3-month-old girls and 4 to 6-month-old girls compared with Adequate Intakes Set by the European Food Safety Authority and US Institute of Medicine

	Boys							Girls									
	0–3-month olds			4–6-month olds				0–3-month olds			4–6-month olds				EFSA AI	US AI	Prevalence of inadequate intake
Study visit	2	4	8	2	4	8	16	2	4	8	2	4	8	16			
Calcium, mg	551	616	694	618	589	678	694	513	497	507	560	608	581	585	200	200	Low
Phosphorus, mg	390	436	491	438	417	480	491	363	352	359	396	431	411	414	100	100	Low
Iron, mg	7.0	7.8	8.8	7.8	7.4	8.6	8.8	6.5	6.3	6.4	9.0	9.7	9.3	9.4	0.3	0.27	Low
Chloride, mg	379	424	477	425	405	467	477	353	342	349	385	418	399	402	300	180	Low
Sodium, mg	185	207	233	208	198	228	233	172	167	170	188	205	195	197	120	120	Low
Potassium, mg	517	578	651	580	552	636	651	481	466	475	525	570	545	549	400	400	Low
Magnesium, mg	50	56	63	56	54	62	63	47	45	46	51	55	53	53	25	30	Low

AI = adequate intake; EFSA = European Food Safety Authority.
