# Supplementary material for: Mineral Intake and Status of Cow's Milk Allergic Infants Consuming an Amino Acid-based Formula
Source: J Pediatr Gastroenterol Nutr. 2017 Aug 22;65(3):346–9. doi: 10.1097/MPG.0000000000001655 (PMC5559186; doi:10.1097/MPG.0000000000001655)
Supplement: Supplemental Digital Content [file jpga-65-346-s001.docx]

**Supplemental Table 1. Macronutrient and mineral profile of study products per 100 kcal of prepared product**

|  | Neocate with synbiotics | Neocate without synbiotics |
| --- | --- | --- |
| Energy | 67 kcal/100 ml | 67 kcal/100 ml |
| Macronutrients |  |  |
| Proteins, g | 2.8 | 3.1 |
| Carbohydrates, g | 11.3 | 11.7 |
| Fats, g | 4.8 | 4.5 |
| Dietary Fiber, g | 1.1 | - |
| Bifidobacterium breve M16-V, CFU | 2.11x10^9^ | - |
| Minerals |  |  |
| Sodium, mg | 40 | 37 |
| Potassium, mg | 105 | 155 |
| Chloride, mg | 75 | 77 |
| Calcium, mg | 90 | 124 |
| Phosphorus, mg | 63 | 93 |
| Magnesium, mg | 11.2 | 12.4 |
| Iron, mg | 1.5 | 1.9 |
| Zinc, mg | 1.1 | 1.7 |
| Copper, µg | 76 | 124 |
| Manganese, µg | 76 | 90 |
| Selenium, µg | 2.8 | 3.7 |
| Iodine, µg | 17.5 | 15.4 |
