# Supplementary material for: Mineral Intake and Status of Cow's Milk Allergic Infants Consuming an Amino Acid-based Formula
Source: J Pediatr Gastroenterol Nutr. 2017 Aug 22;65(3):346–9. doi: 10.1097/MPG.0000000000001655 (PMC5559186; doi:10.1097/MPG.0000000000001655)
Supplement: Supplemental Digital Content [file jpga-65-346-s002.docx]

**Supplemental Table 2. Diagnostic criteria for cow’s milk allergy used in the present study**

|  |
| --- |
| Positive double-blind placebo-controlled food challenge with cow’s milk |
| A confirmed history of acute severe reaction after isolated accidental ingestion with a positive test result for IgE antibody |
| Confirmed history of a reaction to cow milk protein with CM-specific IgE of >15kU/L for children over two years and > 5kU/L for children under two years |
| Confirmed clinical history of a reaction to cow milk protein with a positive skin prick test with a resulting wheal diameter greater or equal to 3mm |
| No confirmed clinical history of a reaction to cow milk protein subjects with a positive skin prick test with a resulting wheal diameter greater or equal to 8mm for children over 2 years and greater or equal to 6mm for children under 2 years |
| For children with an Allergic Eosinophilic Gastroenteritis (AEG), documentation of eosinophilic infiltration and resolution of symptoms on a diet that restricted cow milk (and other foods) with reoccurrence after re-administration of cow milk |
| For infants up to 8 months of age with a non-IgE mediated CMA (including food protein induced enterocolitis, food protein induced proctitis, gastroesophageal reflux disease, food protein induced enteropathy, etc) documentation of symptoms suggestive of non-IgE mediated CMA and resolution of symptoms on a diet that restricted cow milk (and other foods) with reoccurrence after re-administration of cow milk. |
