# Supplementary material for: Mineral Intake and Status of Cow's Milk Allergic Infants Consuming an Amino Acid-based Formula
Source: J Pediatr Gastroenterol Nutr. 2017 Aug 22;65(3):346–9. doi: 10.1097/MPG.0000000000001655 (PMC5559186; doi:10.1097/MPG.0000000000001655)
Supplement: Supplemental Digital Content [file jpga-65-346-s003.docx]

**Supplemental Table 3**. Baseline characteristics of all subjects (n=110)

|  | Mean ± SD or % |
| --- | --- |
| Age (months) | 4.55 ± 2.42 |
| Sex, male (%) | 61.8 |
| Ethnicity (%) |  |
| Asian | 4 |
| Black | 14 |
| Latino | 13 |
| White | 62 |
| Other | 7 |
| Weight (kg) | 6.54 ± 1.50 |
| Length (cm) | 63.08 ± 5.42 |
| Nutritional formula prior to study entry (%) |  |
| AAF | 57 |
| eHF | 8 |
| Soy-based formula | 31 |
| Other/none | 4 |
| Confirmed IgE-med CMA (%) | 33 |

AAF: amino acid-based formula; eHF: extensively hydrolysed formula; IgE-med CMA: IgE-mediated cow’s milk allergy
