# Supplementary figures and images for: Mineral Intake and Status of Cow's Milk Allergic Infants Consuming an Amino Acid-based Formula
Source: J Pediatr Gastroenterol Nutr. 2017 Aug 22;65(3):346–9. doi: 10.1097/MPG.0000000000001655 (PMC5559186; doi:10.1097/MPG.0000000000001655)

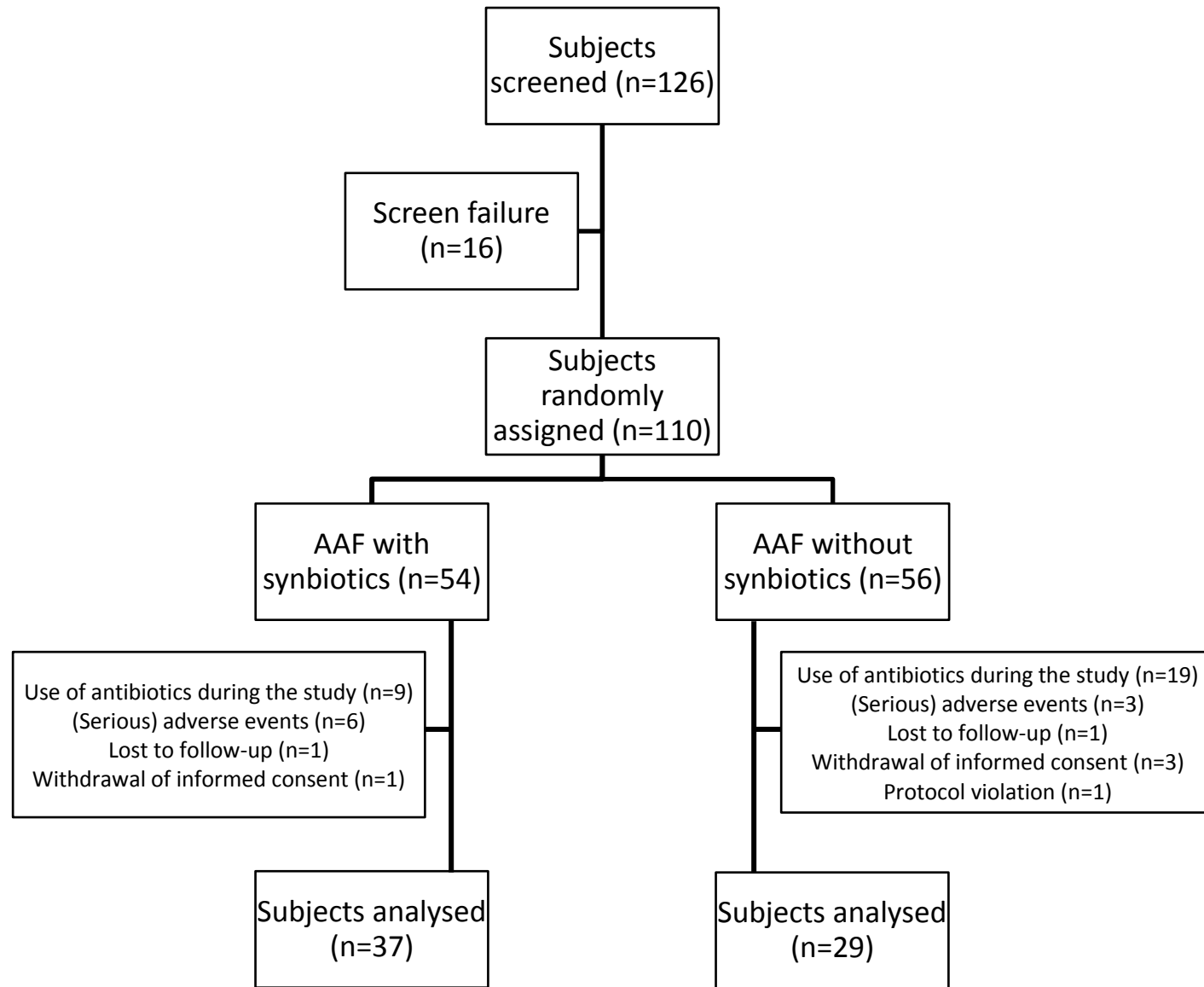

**Supplemental Figure 1.** Study flow chart. AAF: amino acid-based formula

Supplement: Supplemental Digital Content [file jpga-65-346-s004.pdf]
