# Supplementary material for: Mineral Intake and Status of Cow's Milk Allergic Infants Consuming an Amino Acid-based Formula
Source: J Pediatr Gastroenterol Nutr. 2017 Aug 22;65(3):346–9. doi: 10.1097/MPG.0000000000001655 (PMC5559186; doi:10.1097/MPG.0000000000001655)
Supplement: Supplemental Digital Content [file jpga-65-346-s005.docx]

**Supplemental Table 4. Energy intake (p5, p25, p50, p75 and p95) from formula alone compared to Dietary Reference Values for energy**

| **Study visit (wks)** | **Energy intake (kcal/d) from formula alone in boys** | | | | | | | | | | | | | | | | | | | |
| --- | --- | --- | --- | --- | --- | --- | --- | --- | --- | --- | --- | --- | --- | --- | --- | --- | --- | --- | --- | --- |
|  | **0 – 3 months** | | | | | **4 – 6 months** | | | | | **7 – 9 months** | | | | | **10 – 12 months** | | | | |
|  | P5 | P25 | P50 | P75 | P95 | P5 | P25 | P50 | P75 | P95 | P5 | P25 | P50 | P75 | P95 | P5 | P25 | P50 | P75 | P95 |
| 2 | 356 | 408 | 467 | 552 | 611 | 348 | 483 | 524 | 555 | 729 | 322 | 389 | 450 | 536 | 781 |  |  |  |  |  |
| 4 | 390 | 419 | 522 | 581 | 670 | 357 | 421 | 499 | 573 | 674 | 325 | 426 | 482 | 655 | 902 |  |  |  |  |  |
| 8 | 502 | 581 | 588 | 621 | 689 | 336 | 419 | 575 | 610 | 687 | 256 | 423 | 522 | 617 | 798 | 313 | 335 | 413 | 639 | 658 |
| 16 |  |  |  |  |  | 297 | 443 | 588 | 660 | 793 | 404 | 461 | 527 | 594 | 666 | 237 | 428 | 527 | 629 | 645 |
| ***UK EAR* ^1, 2^** | 584 | | | | | 611 | | | | | 679 | | | | | 756 | | | | |
| ***US EER* ^1, 2^** | 537 | | | | | 596 | | | | | 708 | | | | | 818 | | | | |

| **Study visit (wks)** | **Energy intake (kcal/d) from formula alone in girls** | | | | | | | | | | | | | | | | | | | |
| --- | --- | --- | --- | --- | --- | --- | --- | --- | --- | --- | --- | --- | --- | --- | --- | --- | --- | --- | --- | --- |
|  | **0 – 3 months** | | | | | **4 – 6 months** | | | | | **7 – 9 months** | | | | | **10 – 12 months** | | | | |
|  | P5 | P25 | P50 | P75 | P95 | P5 | P25 | P50 | P75 | P95 | P5 | P25 | P50 | P75 | P95 | P5 | P25 | P50 | P75 | P95 |
| 2 | 336 | 397 | 434 | 467 | 537 | 243 | 336 | 474 | 579 | 1142 | 298 | 428 | 509 | 592 | 594 |  |  |  |  |  |
| 4 | 265 | 395 | 421 | 467 | 569 | 374 | 468 | 515 | 641 | 698 | 294 | 428 | 472 | 504 | 645 |  |  |  |  |  |
| 8 | 400 | 413 | 429 | 654 | 833 | 309 | 383 | 492 | 535 | 597 | 293 | 436 | 476 | 560 | 726 | 321 | 443 | 515 | 568 | 874 |
| 16 |  |  |  |  |  | 334 | 406 | 495 | 517 | 801 | 355 | 424 | 538 | 655 | 1047 | 470 | 634 | 641 | 647 | 700 |
| ***UK EAR* ^1, 2^** | 527 | | | | | 569 | | | | | 622 | | | | | 692 | | | | |
| ***US EER* ^1, 2^** | 486 | | | | | 551 | | | | | 643 | | | | | 742 | | | | |

EAR, Estimated Average Requirement set by the UK Scientific Advisory Committee on Nutrition; EER, Estimated Energy Requirement set by the US Institute of Medicine; ^1^Due to correlation between energy intake and energy requirement adequacy cannot be assessed, ^2^ Mean of specific age groups, e.g. EAR of 4 – 6 months is calculated as the average of the EAR of 4, 5 and 6 months.
