# Supplementary material for: Mineral Intake and Status of Cow's Milk Allergic Infants Consuming an Amino Acid-based Formula
Source: J Pediatr Gastroenterol Nutr. 2017 Aug 22;65(3):346–9. doi: 10.1097/MPG.0000000000001655 (PMC5559186; doi:10.1097/MPG.0000000000001655)
Supplement: Supplemental Digital Content [file jpga-65-346-s006.docx]

**Supplemental Table 5. Mineral intakes (p5, p25, p50, p75 and p95) from formula alone and formula plus complementary foods versus Adequate Intakes**

Table 5a. Calcium intake (p5, p25, p50, p75 and p95) from formula alone and from formula plus complementary alone compared to Adequate Intakes (AI) set by the European Food Safety Authority (EFSA) and US Institute of Medicine

| **Study visit (wks)** | **Calcium intake (mg/d) from formula alone in boys** | | | | | | | | | | | | | | | | | | | |
| --- | --- | --- | --- | --- | --- | --- | --- | --- | --- | --- | --- | --- | --- | --- | --- | --- | --- | --- | --- | --- |
|  | **0 – 3 months** | | | | | **4 – 6 months** | | | | | **7 – 9 months** | | | | | **10 – 12 months** | | | | |
|  | P5 | P25 | P50 | P75 | P95 | P5 | P25 | P50 | P75 | P95 | P5 | P25 | P50 | P75 | P95 | P5 | P25 | P50 | P75 | P95 |
| 2 | 420 | 482 | 551 | 652 | 722 | 410 | 571 | 618 | 655 | 861 | 380 | 459 | 531 | 633 | 922 |  |  |  |  |  |
| 4 | 460 | 495 | 616 | 686 | 791 | 422 | 497 | 589 | 676 | 795 | 384 | 503 | 569 | 774 | 1065 |  |  |  |  |  |
| 8 | 593 | 686 | 694 | 733 | 814 | 397 | 494 | 678 | 720 | 811 | 302 | 499 | 616 | 728 | 942 | 370 | 396 | 487 | 754 | 777 |
| 16 |  |  |  |  |  | 351 | 522 | 694 | 780 | 936 | 478 | 544 | 622 | 702 | 786 | 280 | 505 | 622 | 743 | 761 |
|  | | | | | | | | | | | | | | | | | | | | |
|  | **Calcium intake (mg/d) from formula plus complementary foods in boys** | | | | | | | | | | | | | | | | | | | |
|  | **0 – 3 months** | | | | | **4 – 6 months** | | | | | **7 – 9 months** | | | | | **10 – 12 months** | | | | |
|  | P5 | P25 | P50 | P75 | P95 | P5 | P25 | P50 | P75 | P95 | P5 | P25 | P50 | P75 | P95 | P5 | P25 | P50 | P75 | P95 |
| 2 | 420 | 482 | 551 | 652 | 722 | 523 | 684 | 731 | 768 | 974 | 558 | 637 | 709 | 811 | 1100 |  |  |  |  |  |
| 4 | 460 | 495 | 616 | 686 | 791 | 535 | 610 | 702 | 789 | 908 | 562 | 681 | 747 | 952 | 1243 |  |  |  |  |  |
| 8 | 593 | 686 | 694 | 733 | 814 | 510 | 607 | 791 | 833 | 924 | 480 | 677 | 794 | 906 | 1120 | 575 | 601 | 692 | 959 | 982 |
| 16 |  |  |  |  |  | 464 | 635 | 807 | 893 | 1049 | 656 | 722 | 800 | 880 | 964 | 485 | 710 | 827 | 948 | 966 |
|  | | | | | | | | | | | | | | | | | | | | |
| ***EFSA AI*** | 200 | | | | | 200 | | | | | 400 | | | | | 400 | | | | |
| ***US AI*** | 200 | | | | | 200 | | | | | 260 | | | | | 260 | | | | |
| ***Prevalence inadequate intake^1^*** | low | | | | | low | | | | | low | | | | | low | | | | |

| **Study visit (wks)** | | **Calcium intake (mg/d) from formula alone in girls** | | | | | | | | | | | | | | | | | | | |
| --- | --- | --- | --- | --- | --- | --- | --- | --- | --- | --- | --- | --- | --- | --- | --- | --- | --- | --- | --- | --- | --- |
|  |  | **0 – 3 months** | | | | | **4 – 6 months** | | | | | **7 – 9 months** | | | | | **10 – 12 months** | | | | |
|  |  | P5 | P25 | P50 | P75 | P95 | P5 | P25 | P50 | P75 | P95 | P5 | P25 | P50 | P75 | P95 | P5 | P25 | P50 | P75 | P95 |
| 2 | | 396 | 469 | 513 | 551 | 634 | 287 | 396 | 560 | 683 | 1348 | 352 | 505 | 601 | 699 | 701 |  |  |  |  |  |
| 4 | | 313 | 466 | 497 | 552 | 672 | 442 | 553 | 608 | 756 | 824 | 347 | 505 | 557 | 595 | 761 |  |  |  |  |  |
| 8 | | 472 | 487 | 507 | 772 | 984 | 364 | 452 | 581 | 632 | 705 | 436 | 515 | 561 | 661 | 698 | 379 | 522 | 608 | 671 | 1032 |
| 16 | |  |  |  |  |  | 394 | 479 | 585 | 610 | 945 | 419 | 501 | 635 | 773 | 1236 | 555 | 748 | 756 | 764 | 827 |
|  | | | | | | | | | | | | | | | | | | | | | |
|  | **Calcium intake (mg/d) from formula plus complementary foods in girls** | | | | | | | | | | | | | | | | | | | | |
|  | **0 – 3 months** | | | | | | **4 – 6 months** | | | | | **7 – 9 months** | | | | | **10 – 12 months** | | | | |
|  | P5 | | P25 | P50 | P75 | P95 | P5 | P25 | P50 | P75 | P95 | P5 | P25 | P50 | P75 | P95 | P5 | P25 | P50 | P75 | P95 |
| 2 | 396 | | 469 | 513 | 551 | 634 | 400 | 509 | 673 | 796 | 1461 | 530 | 683 | 779 | 877 | 879 |  |  |  |  |  |
| 4 | 313 | | 466 | 497 | 552 | 672 | 555 | 666 | 721 | 869 | 937 | 525 | 683 | 735 | 773 | 939 |  |  |  |  |  |
| 8 | 472 | | 487 | 507 | 772 | 984 | 477 | 565 | 694 | 745 | 818 | 614 | 693 | 739 | 839 | 876 | 584 | 727 | 813 | 876 | 1237 |
| 16 |  | |  |  |  |  | 507 | 592 | 698 | 723 | 1058 | 597 | 679 | 813 | 951 | 1414 | 760 | 953 | 961 | 969 | 1032 |
|  | | | | | | | | | | | | | | | | | | | | | |
| ***EFSA AI*** | 200 | | | | | | 200 | | | | | 400 | | | | | 400 | | | | |
| ***US AI*** | 200 | | | | | | 200 | | | | | 260 | | | | | 260 | | | | |
| ***Prevalence inadequate intake^1^*** | low | | | | | | low | | | | | low | | | | | low | | | | |

^1^Prevalence of inadequate intake: low = when the median intake is above the AI the prevalence of inadequate intake is likely to be low

Table 5b. Phosphorus intake (p5, p25, p50, p75 and p95) from formula alone compared to Adequate Intakes (AI) set by the European Food Safety Authority (EFSA) and US Institute of Medicine

| **Study visit (wks)** | **Phosphorus intake (mg/d) from formula alone in boys** | | | | | | | | | | | | | | | | | | | |
| --- | --- | --- | --- | --- | --- | --- | --- | --- | --- | --- | --- | --- | --- | --- | --- | --- | --- | --- | --- | --- |
|  | **0 – 3 months** | | | | | **4 – 6 months** | | | | | **7 – 9 months** | | | | | **10 – 12 months** | | | | |
|  | P5 | P25 | P50 | P75 | P95 | P5 | P25 | P50 | P75 | P95 | P5 | P25 | P50 | P75 | P95 | P5 | P25 | P50 | P75 | P95 |
| 2 | 297 | 341 | 390 | 462 | 511 | 291 | 404 | 438 | 464 | 610 | 269 | 325 | 376 | 448 | 653 |  |  |  |  |  |
| 4 | 326 | 351 | 436 | 486 | 560 | 299 | 352 | 417 | 479 | 563 | 272 | 356 | 403 | 548 | 754 |  |  |  |  |  |
| 8 | 419 | 486 | 491 | 519 | 576 | 281 | 350 | 480 | 510 | 574 | 214 | 353 | 436 | 515 | 667 | 262 | 280 | 345 | 534 | 550 |
| 16 |  |  |  |  |  | 248 | 370 | 491 | 552 | 662 | 338 | 385 | 440 | 497 | 557 | 198 | 357 | 440 | 526 | 539 |
|  | | | | | | | | | | | | | | | | | | | | |
| ***EFSA AI*** | 100 | | | | | 100 | | | | | 300 | | | | | 300 | | | | |
| ***US AI*** | 100 | | | | | 100 | | | | | 275 | | | | | 275 | | | | |
| ***Prevalence inadequate intake^1^*** | low | | | | | low | | | | | low | | | | | low | | | | |

| **Study visit (wks)** | **Phosphorus intake (mg/d) from formula alone in girls** | | | | | | | | | | | | | | | | | | | |
| --- | --- | --- | --- | --- | --- | --- | --- | --- | --- | --- | --- | --- | --- | --- | --- | --- | --- | --- | --- | --- |
|  | **0 – 3 months** | | | | | **4 – 6 months** | | | | | **7 – 9 months** | | | | | **10 – 12 months** | | | | |
|  | P5 | P25 | P50 | P75 | P95 | P5 | P25 | P50 | P75 | P95 | P5 | P25 | P50 | P75 | P95 | P5 | P25 | P50 | P75 | P95 |
| 2 | 281 | 332 | 363 | 390 | 449 | 203 | 281 | 396 | 484 | 955 | 249 | 358 | 426 | 495 | 496 |  |  |  |  |  |
| 4 | 222 | 330 | 352 | 391 | 476 | 313 | 391 | 431 | 535 | 582 | 246 | 357 | 395 | 422 | 539 |  |  |  |  |  |
| 8 | 334 | 345 | 359 | 546 | 697 | 258 | 320 | 411 | 447 | 499 | 309 | 364 | 397 | 468 | 494 | 268 | 270 | 431 | 475 | 731 |
| 16 |  |  |  |  |  | 279 | 339 | 414 | 432 | 669 | 297 | 355 | 450 | 547 | 875 | 393 | 530 | 535 | 541 | 585 |
|  | | | | | | | | | | | | | | | | | | | | |
| ***EFSA AI*** | 100 | | | | | 100 | | | | | 300 | | | | | 300 | | | | |
| ***US AI*** | 100 | | | | | 100 | | | | | 275 | | | | | 275 | | | | |
| ***Prevalence inadequate intake^1^*** | low | | | | | low | | | | | low | | | | | low | | | | |

^1^Prevalence of inadequate intake: low = when the median intake is above the AI the prevalence of inadequate intake is likely to be low

Table 5c. Iron intake (p5, p25, p50, p75 and p95) from formula alone and from formula plus complementary alone compared to Adequate Intakes (AI) set by the European Food Safety Authority (EFSA) and US Institute of Medicine

| **Study visit (wks)** | **Iron intake (mg/d) from formula alone in boys** | | | | | | | | | | | | | | | | | | | |
| --- | --- | --- | --- | --- | --- | --- | --- | --- | --- | --- | --- | --- | --- | --- | --- | --- | --- | --- | --- | --- |
|  | **0 – 3 months** | | | | | **4 – 6 months** | | | | | **7 – 9 months** | | | | | **10 – 12 months** | | | | |
|  | P5 | P25 | P50 | P75 | P95 | P5 | P25 | P50 | P75 | P95 | P5 | P25 | P50 | P75 | P95 | P5 | P25 | P50 | P75 | P95 |
| 2 | 5.3 | 6.1 | 7.0 | 8.2 | 9.1 | 5.2 | 7.2 | 7.8 | 8.3 | 10.9 | 4.8 | 5.8 | 6.7 | 8.0 | 11.7 |  |  |  |  |  |
| 4 | 5.8 | 6.3 | 7.8 | 8.7 | 10.0 | 5.3 | 6.3 | 7.4 | 8.6 | 10.1 | 4.8 | 6.4 | 7.2 | 9.8 | 13.5 |  |  |  |  |  |
| 8 | 7.5 | 8.7 | 8.8 | 9.3 | 10.3 | 5.0 | 6.2 | 8.6 | 9.1 | 10.3 | 3.8 | 6.3 | 7.8 | 9.2 | 11.9 | 4.7 | 5.0 | 6.2 | 9.5 | 9.8 |
| 16 |  |  |  |  |  | 4.4 | 6.6 | 8.8 | 9.9 | 11.8 | 6.0 | 6.9 | 7.9 | 8.9 | 9.9 | 3.5 | 6.4 | 7.9 | 9.4 | 9.6 |
|  | | | | | | | | | | | | | | | | | | | | |
|  | **Iron intake (mg/d) from formula plus complementary foods in boys** | | | | | | | | | | | | | | | | | | | |
|  | **0 – 3 months** | | | | | **4 – 6 months** | | | | | **7 – 9 months** | | | | | **10 – 12 months** | | | | |
|  | P5 | P25 | P50 | P75 | P95 | P5 | P25 | P50 | P75 | P95 | P5 | P25 | P50 | P75 | P95 | P5 | P25 | P50 | P75 | P95 |
| 2 | 5.3 | 6.1 | 7.0 | 8.2 | 9.1 | 7.2 | 9.2 | 9.8 | 10.2 | 12.9 | 8.3 | 9.3 | 10.2 | 11.5 | 15.2 |  |  |  |  |  |
| 4 | 5.8 | 6.3 | 7.8 | 8.7 | 10.0 | 7.3 | 8.3 | 9.4 | 10.5 | 12.0 | 8.3 | 9.9 | 10.7 | 13.3 | 17.0 |  |  |  |  |  |
| 8 | 7.5 | 8.7 | 8.8 | 9.3 | 10.3 | 7.0 | 8.2 | 10.5 | 11.1 | 12.2 | 7.3 | 9.8 | 11.3 | 12.7 | 15.4 | 9.0 | 9.3 | 10.5 | 13.8 | 14.1 |
| 16 |  |  |  |  |  | 6.4 | 8.6 | 10.7 | 11.8 | 13.8 | 9.5 | 10.4 | 11.4 | 12.4 | 13.4 | 7.9 | 10.7 | 12.2 | 13.7 | 13.9 |
|  | | | | | | | | | | | | | | | | | | | | |
| ***EFSA AI*** | 0.3 | | | | | 0.3 | | | | | 8 | | | | | 8 | | | | |
| ***US AI*** | 0.27 | | | | | 0.27 | | | | | 11 | | | | | 11 | | | | |
| ***Prevalence inadequate intake^1^*** | low | | | | | low | | | | | low/no statement | | | | | low/no statement | | | | |

| **Study visit (wks)** | | **Iron intake (mg/d) from formula alone in girls** | | | | | | | | | | | | | | | | | | | |
| --- | --- | --- | --- | --- | --- | --- | --- | --- | --- | --- | --- | --- | --- | --- | --- | --- | --- | --- | --- | --- | --- |
|  |  | **0 – 3 months** | | | | | **4 – 6 months** | | | | | **7 – 9 months** | | | | | **10 – 12 months** | | | | |
|  |  | P5 | P25 | P50 | P75 | P95 | P5 | P25 | P50 | P75 | P95 | P5 | P25 | P50 | P75 | P95 | P5 | P25 | P50 | P75 | P95 |
| 2 | | 5.0 | 5.9 | 6.5 | 7.0 | 8.0 | 3.6 | 5.0 | 7.1 | 8.6 | 17.0 | 4.4 | 6.4 | 7.6 | 8.8 | 8.9 |  |  |  |  |  |
| 4 | | 4.0 | 5.9 | 6.3 | 7.0 | 8.5 | 5.6 | 7.0 | 7.7 | 9.6 | 10.4 | 4.4 | 6.4 | 7.0 | 7.5 | 9.6 |  |  |  |  |  |
| 8 | | 6.0 | 6.2 | 6.4 | 9.8 | 12.4 | 4.6 | 5.7 | 7.3 | 8.0 | 8.9 | 5.5 | 6.5 | 7.1 | 8.4 | 8.8 | 4.8 | 6.6 | 7.7 | 8.5 | 13.0 |
| 16 | |  |  |  |  |  | 5.0 | 6.1 | 7.4 | 7.4 | 12.0 | 5.3 | 6.3 | 8.0 | 9.8 | 15.6 | 7.0 | 9.5 | 9.6 | 9.7 | 10.5 |
|  | | | | | | | | | | | | | | | | | | | | | |
|  | **Iron intake (mg/d) from formula plus complementary foods in girls** | | | | | | | | | | | | | | | | | | | | |
|  | **0 – 3 months** | | | | | | **4 – 6 months** | | | | | **7 – 9 months** | | | | | **10 – 12 months** | | | | |
|  | P5 | | P25 | P50 | P75 | P95 | P5 | P25 | P50 | P75 | P95 | P5 | P25 | P50 | P75 | P95 | P5 | P25 | P50 | P75 | P95 |
| 2 | 5.0 | | 5.9 | 6.5 | 7.0 | 8.0 | 5.6 | 7.0 | 9.0 | 10.6 | 19.0 | 7.9 | 9.9 | 11.1 | 12.3 | 12.4 |  |  |  |  |  |
| 4 | 4.0 | | 5.9 | 6.3 | 7.0 | 8.5 | 7.6 | 9.0 | 9.7 | 11.5 | 12.4 | 7.9 | 9.9 | 10.5 | 11.0 | 13.1 |  |  |  |  |  |
| 8 | 6.0 | | 6.2 | 6.4 | 9.8 | 12.4 | 6.6 | 7.7 | 9.3 | 10.0 | 10.9 | 9.0 | 10.0 | 10.6 | 11.9 | 12.3 | 9.1 | 10.9 | 12.0 | 12.8 | 17.4 |
| 16 |  | |  |  |  |  | 6.9 | 8.0 | 9.4 | 9.7 | 13.9 | 8.8 | 9.8 | 11.5 | 13.3 | 19.1 | 11.3 | 13.8 | 13.9 | 14.0 | 14.8 |
|  | | | | | | | | | | | | | | | | | | | | | |
| ***EFSA AI*** | | 0.3 | | | | | 0.3 | | | | | 8 | | | | | 8 | | | | |
| ***US AI*** | | 0.27 | | | | | 0.27 | | | | | 11 | | | | | 11 | | | | |
| ***Prevalence inadequate intake^1^*** | | low | | | | | low | | | | | low/no statement | | | | | low/no statement | | | | |

^1^Prevalence of inadequate intake: low = when the median intake is above the AI the prevalence of inadequate intake is likely to be low; low/no statement = when the median intake is above or below the AI depending on the reference for the AI and/or study visit

Table 5d. Chloride intake (p5, p25, p50, p75 and p95) from formula alone compared to Adequate Intakes (AI) set by the European Food Safety Authority (EFSA) and US Institute of Medicine

| **Study visit (wks)** | **Chloride intake (mg/d) from formula alone in boys** | | | | | | | | | | | | | | | | | | | |
| --- | --- | --- | --- | --- | --- | --- | --- | --- | --- | --- | --- | --- | --- | --- | --- | --- | --- | --- | --- | --- |
|  | **0 – 3 months** | | | | | **4 – 6 months** | | | | | **7 – 9 months** | | | | | **10 – 12 months** | | | | |
|  | P5 | P25 | P50 | P75 | P95 | P5 | P25 | P50 | P75 | P95 | P5 | P25 | P50 | P75 | P95 | P5 | P25 | P50 | P75 | P95 |
| 2 | 289 | 332 | 379 | 448 | 496 | 282 | 392 | 425 | 450 | 592 | 261 | 316 | 365 | 435 | 634 |  |  |  |  |  |
| 4 | 316 | 340 | 424 | 472 | 544 | 290 | 342 | 405 | 465 | 547 | 264 | 346 | 391 | 532 | 733 |  |  |  |  |  |
| 8 | 407 | 472 | 477 | 504 | 560 | 273 | 340 | 467 | 495 | 558 | 208 | 343 | 424 | 501 | 648 | 254 | 272 | 335 | 519 | 534 |
| 16 |  |  |  |  |  | 241 | 359 | 477 | 536 | 644 | 328 | 374 | 428 | 483 | 541 | 193 | 347 | 428 | 511 | 524 |
|  | | | | | | | | | | | | | | | | | | | | |
| ***EFSA AI*** | 300 | | | | | 300 | | | | | 270-570 | | | | | 270-570 | | | | |
| ***US AI*** | 180 | | | | | 180 | | | | | 570 | | | | | 570 | | | | |
| ***Prevalence inadequate intake^1^*** | low | | | | | low | | | | | low/no statement | | | | | low/no statement | | | | |

| **Study visit (wks)** | **Chloride intake (mg/d) from formula alone in girls** | | | | | | | | | | | | | | | | | | | |
| --- | --- | --- | --- | --- | --- | --- | --- | --- | --- | --- | --- | --- | --- | --- | --- | --- | --- | --- | --- | --- |
|  | **0 – 3 months** | | | | | **4 – 6 months** | | | | | **7 – 9 months** | | | | | **10 – 12 months** | | | | |
|  | P5 | P25 | P50 | P75 | P95 | P5 | P25 | P50 | P75 | P95 | P5 | P25 | P50 | P75 | P95 | P5 | P25 | P50 | P75 | P95 |
| 2 | 273 | 322 | 353 | 379 | 436 | 198 | 273 | 385 | 470 | 927 | 242 | 348 | 414 | 481 | 482 |  |  |  |  |  |
| 4 | 215 | 320 | 342 | 379 | 462 | 304 | 380 | 418 | 520 | 567 | 239 | 347 | 383 | 410 | 523 |  |  |  |  |  |
| 8 | 324 | 335 | 349 | 531 | 677 | 251 | 311 | 399 | 434 | 485 | 300 | 354 | 386 | 454 | 480 | 261 | 359 | 418 | 461 | 710 |
| 16 |  |  |  |  |  | 271 | 330 | 402 | 420 | 650 | 289 | 345 | 437 | 532 | 850 | 382 | 515 | 520 | 526 | 569 |
|  | | | | | | | | | | | | | | | | | | | | |
| ***EFSA AI*** | 300 | | | | | 300 | | | | | 270-570 | | | | | 270-570 | | | | |
| ***US AI*** | 180 | | | | | 180 | | | | | 570 | | | | | 570 | | | | |
| ***Prevalence inadequate intake^1^*** | low | | | | | low | | | | | low/no statement | | | | | low/no statement | | | | |

^1^Prevalence of inadequate intake: low = when the median intake is above the AI the prevalence of inadequate intake is likely to be low; low/no statement = when the median intake is above or below the AI depending on the reference for the AI and/or study visit

Table 5e. Sodium intake (p5, p25, p50, p75 and p95) from formula alone and from formula plus complementary alone compared to Adequate Intakes (AI) set by the European Food Safety Authority (EFSA) and US Institute of Medicine

| **Study visit (wks)** | **Sodium intake (mg/d) from formula alone in boys** | | | | | | | | | | | | | | | | | | | |
| --- | --- | --- | --- | --- | --- | --- | --- | --- | --- | --- | --- | --- | --- | --- | --- | --- | --- | --- | --- | --- |
|  | **0 – 3 months** | | | | | **4 – 6 months** | | | | | **7 – 9 months** | | | | | **10 – 12 months** | | | | |
|  | P5 | P25 | P50 | P75 | P95 | P5 | P25 | P50 | P75 | P95 | P5 | P25 | P50 | P75 | P95 | P5 | P25 | P50 | P75 | P95 |
| 2 | 141 | 162 | 185 | 219 | 243 | 138 | 192 | 208 | 220 | 290 | 128 | 155 | 179 | 213 | 310 |  |  |  |  |  |
| 4 | 155 | 166 | 207 | 231 | 266 | 142 | 167 | 198 | 227 | 267 | 129 | 169 | 191 | 260 | 358 |  |  |  |  |  |
| 8 | 199 | 231 | 233 | 246 | 274 | 133 | 166 | 228 | 242 | 273 | 101 | 168 | 207 | 245 | 317 | 124 | 133 | 164 | 254 | 261 |
| 16 |  |  |  |  |  | 118 | 176 | 233 | 262 | 315 | 161 | 183 | 209 | 236 | 264 | 94 | 170 | 209 | 250 | 256 |
|  | | | | | | | | | | | | | | | | | | | | |
|  | **Sodium intake (mg/d) from formula plus complementary foods in boys** | | | | | | | | | | | | | | | | | | | |
|  | **0 – 3 months** | | | | | **4 – 6 months** | | | | | **7 – 9 months** | | | | | **10 – 12 months** | | | | |
|  | P5 | P25 | P50 | P75 | P95 | P5 | P25 | P50 | P75 | P95 | P5 | P25 | P50 | P75 | P95 | P5 | P25 | P50 | P75 | P95 |
| 2 | 141 | 162 | 185 | 219 | 243 | 219 | 273 | 289 | 301 | 371 | 373 | 400 | 424 | 458 | 555 |  |  |  |  |  |
| 4 | 155 | 166 | 207 | 231 | 266 | 223 | 248 | 279 | 308 | 348 | 374 | 414 | 436 | 505 | 603 |  |  |  |  |  |
| 8 | 199 | 231 | 233 | 246 | 274 | 214 | 247 | 309 | 323 | 354 | 346 | 413 | 452 | 490 | 562 | 529 | 538 | 569 | 659 | 666 |
| 16 |  |  |  |  |  | 199 | 257 | 314 | 343 | 396 | 406 | 428 | 454 | 481 | 509 | 499 | 575 | 614 | 655 | 661 |
|  | | | | | | | | | | | | | | | | | | | | |
| ***EFSA AI*** | 120 | | | | | 120 | | | | | 170-370 | | | | | 170-370 | | | | |
| ***US AI*** | 120 | | | | | 120 | | | | | 370 | | | | | 370 | | | | |
| ***Prevalence inadequate intake^1^*** | low | | | | | low | | | | | low | | | | | low | | | | |

| **Study visit (wks)** | **Sodium intake (mg/d) from formula alone in girls** | | | | | | | | | | | | | | | | | | | |
| --- | --- | --- | --- | --- | --- | --- | --- | --- | --- | --- | --- | --- | --- | --- | --- | --- | --- | --- | --- | --- |
|  | **0 – 3 months** | | | | | **4 – 6 months** | | | | | **7 – 9 months** | | | | | **10 – 12 months** | | | | |
|  | P5 | P25 | P50 | P75 | P95 | P5 | P25 | P50 | P75 | P95 | P5 | P25 | P50 | P75 | P95 | P5 | P25 | P50 | P75 | P95 |
| 2 | 133 | 158 | 172 | 185 | 213 | 97 | 133 | 188 | 230 | 453 | 118 | 170 | 202 | 235 | 236 |  |  |  |  |  |
| 4 | 105 | 157 | 167 | 185 | 226 | 149 | 186 | 205 | 254 | 277 | 117 | 170 | 187 | 200 | 256 |  |  |  |  |  |
| 8 | 159 | 164 | 170 | 260 | 331 | 123 | 152 | 195 | 212 | 237 | 147 | 173 | 189 | 222 | 235 | 127 | 176 | 205 | 225 | 347 |
| 16 |  |  |  |  |  | 132 | 161 | 197 | 205 | 318 | 141 | 168 | 214 | 260 | 416 | 187 | 252 | 254 | 257 | 278 |
|  | | | | | | | | | | | | | | | | | | | | |
|  | **Sodium intake (mg/d) from formula plus complementary foods in girls** | | | | | | | | | | | | | | | | | | | |
|  | **0 – 3 months** | | | | | **4 – 6 months** | | | | | **7 – 9 months** | | | | | **10 – 12 months** | | | | |
|  | P5 | P25 | P50 | P75 | P95 | P5 | P25 | P50 | P75 | P95 | P5 | P25 | P50 | P75 | P95 | P5 | P25 | P50 | P75 | P95 |
| 2 | 133 | 158 | 172 | 185 | 213 | 178 | 214 | 269 | 311 | 534 | 363 | 415 | 447 | 480 | 481 |  |  |  |  |  |
| 4 | 105 | 157 | 167 | 185 | 226 | 230 | 267 | 285 | 335 | 358 | 362 | 415 | 432 | 445 | 501 |  |  |  |  |  |
| 8 | 159 | 164 | 170 | 260 | 331 | 203 | 233 | 276 | 293 | 318 | 392 | 418 | 434 | 467 | 480 | 532 | 581 | 610 | 630 | 752 |
| 16 |  |  |  |  |  | 213 | 242 | 278 | 286 | 399 | 386 | 413 | 459 | 505 | 661 | 592 | 657 | 659 | 662 | 683 |
|  | | | | | | | | | | | | | | | | | | | | |
| ***EFSA AI*** | 120 | | | | | 120 | | | | | 170-370 | | | | | 170-370 | | | | |
| ***US AI*** | 120 | | | | | 120 | | | | | 370 | | | | | 370 | | | | |
| ***Prevalence inadequate intake^1^*** | low | | | | | low | | | | | low | | | | | low | | | | |

^1^Prevalence of inadequate intake: low = when the median intake is above the AI the prevalence of inadequate intake is likely to be low

Table 5f. Potassium intake (p5, p25, p50, p75 and p95) from formula alone compared to Adequate Intakes (AI) set by the European Food Safety Authority (EFSA) and US Institute of Medicine

| **Study visit (wks)** | **Potassium intake (mg/d) from formula alone in boys** | | | | | | | | | | | | | | | | | | | |
| --- | --- | --- | --- | --- | --- | --- | --- | --- | --- | --- | --- | --- | --- | --- | --- | --- | --- | --- | --- | --- |
|  | **0 – 3 months** | | | | | **4 – 6 months** | | | | | **7 – 9 months** | | | | | **10 – 12 months** | | | | |
|  | P5 | P25 | P50 | P75 | P95 | P5 | P25 | P50 | P75 | P95 | P5 | P25 | P50 | P75 | P95 | P5 | P25 | P50 | P75 | P95 |
| 2 | 394 | 452 | 517 | 612 | 677 | 385 | 535 | 580 | 614 | 808 | 356 | 431 | 498 | 594 | 865 |  |  |  |  |  |
| 4 | 431 | 466 | 578 | 644 | 742 | 396 | 466 | 552 | 634 | 746 | 360 | 472 | 534 | 726 | 999 |  |  |  |  |  |
| 8 | 556 | 644 | 651 | 687 | 764 | 372 | 464 | 636 | 676 | 761 | 283 | 468 | 578 | 683 | 884 | 347 | 371 | 457 | 708 | 729 |
| 16 |  |  |  |  |  | 329 | 490 | 651 | 731 | 878 | 448 | 510 | 582 | 658 | 738 | 263 | 474 | 583 | 697 | 714 |
|  | | | | | | | | | | | | | | | | | | | | |
| ***EFSA AI*** | 400 | | | | | 400 | | | | | 800 | | | | | 800 | | | | |
| ***US AI*** | 400 | | | | | 400 | | | | | 700 | | | | | 700 | | | | |
| ***Prevalence inadequate intake^1^*** | low | | | | | low | | | | | no statement | | | | | no statement | | | | |

| **Study visit (wks)** | **Potassium intake (mg/d) from formula alone in girls** | | | | | | | | | | | | | | | | | | | |
| --- | --- | --- | --- | --- | --- | --- | --- | --- | --- | --- | --- | --- | --- | --- | --- | --- | --- | --- | --- | --- |
|  | **0 – 3 months** | | | | | **4 – 6 months** | | | | | **7 – 9 months** | | | | | **10 – 12 months** | | | | |
|  | P5 | P25 | P50 | P75 | P95 | P5 | P25 | P50 | P75 | P95 | P5 | P25 | P50 | P75 | P95 | P5 | P25 | P50 | P75 | P95 |
| 2 | 372 | 440 | 481 | 517 | 595 | 270 | 372 | 525 | 641 | 1265 | 330 | 474 | 564 | 656 | 658 |  |  |  |  |  |
| 4 | 294 | 437 | 466 | 517 | 630 | 415 | 519 | 570 | 709 | 773 | 325 | 474 | 523 | 559 | 714 |  |  |  |  |  |
| 8 | 442 | 457 | 475 | 724 | 923 | 342 | 424 | 545 | 592 | 661 | 409 | 483 | 527 | 620 | 655 | 355 | 490 | 570 | 629 | 968 |
| 16 |  |  |  |  |  | 369 | 450 | 549 | 572 | 887 | 394 | 470 | 596 | 725 | 1159 | 521 | 702 | 709 | 717 | 776 |
|  | | | | | | | | | | | | | | | | | | | | |
| ***EFSA AI*** | 400 | | | | | 400 | | | | | 800 | | | | | 800 | | | | |
| ***US AI*** | 400 | | | | | 400 | | | | | 700 | | | | | 700 | | | | |
| ***Prevalence inadequate intake^1^*** | low | | | | | low | | | | | no statement | | | | | no statement | | | | |

^1^Prevalence of inadequate intake: low = when the median intake is above the AI the prevalence of inadequate intake is likely to be low; no statement = when the median intake is below the AI the adequacy cannot be evaluated

Table 5g. Magnesium intake (p5, p25, p50, p75 and p95) from formula alone compared to Adequate Intakes (AI) set by the European Food Safety Authority (EFSA) and US Institute of Medicine

| **Study visit (wks)** | **Magnesium intake (mg/d) from formula alone in boys** | | | | | | | | | | | | | | | | | | | |
| --- | --- | --- | --- | --- | --- | --- | --- | --- | --- | --- | --- | --- | --- | --- | --- | --- | --- | --- | --- | --- |
|  | **0 – 3 months** | | | | | **4 – 6 months** | | | | | **7 – 9 months** | | | | | **10 – 12 months** | | | | |
|  | P5 | P25 | P50 | P75 | P95 | P5 | P25 | P50 | P75 | P95 | P5 | P25 | P50 | P75 | P95 | P5 | P25 | P50 | P75 | P95 |
| 2 | 38 | 44 | 50 | 59 | 66 | 37 | 52 | 56 | 60 | 78 | 35 | 42 | 48 | 58 | 84 |  |  |  |  |  |
| 4 | 42 | 45 | 56 | 62 | 72 | 38 | 45 | 54 | 62 | 72 | 35 | 46 | 52 | 70 | 97 |  |  |  |  |  |
| 8 | 54 | 62 | 63 | 67 | 74 | 36 | 45 | 62 | 66 | 74 | 27 | 45 | 56 | 66 | 86 | 34 | 36 | 44 | 69 | 71 |
| 16 |  |  |  |  |  | 32 | 48 | 63 | 71 | 85 | 43 | 50 | 57 | 64 | 72 | 26 | 46 | 57 | 68 | 69 |
|  | | | | | | | | | | | | | | | | | | | | |
| ***EFSA AI*** | 25 | | | | | 25 | | | | | 80 | | | | | 80 | | | | |
| ***US AI*** | 30 | | | | | 30 | | | | | 75 | | | | | 75 | | | | |
| ***Prevalence inadequate intake^1^*** | low | | | | | low | | | | | no statement | | | | | no statement | | | | |

| **Study visit (wks)** | **Magnesium intake (mg/d) from formula alone in girls** | | | | | | | | | | | | | | | | | | | |
| --- | --- | --- | --- | --- | --- | --- | --- | --- | --- | --- | --- | --- | --- | --- | --- | --- | --- | --- | --- | --- |
|  | **0 – 3 months** | | | | | **4 – 6 months** | | | | | **7 – 9 months** | | | | | **10 – 12 months** | | | | |
|  | P5 | P25 | P50 | P75 | P95 | P5 | P25 | P50 | P75 | P95 | P5 | P25 | P50 | P75 | P95 | P5 | P25 | P50 | P75 | P95 |
| 2 | 36 | 43 | 47 | 50 | 58 | 26 | 36 | 51 | 62 | 123 | 32 | 46 | 55 | 64 | 64 |  |  |  |  |  |
| 4 | 28 | 42 | 45 | 50 | 61 | 40 | 50 | 55 | 69 | 75 | 32 | 46 | 51 | 54 | 69 |  |  |  |  |  |
| 8 | 43 | 44 | 46 | 70 | 90 | 33 | 41 | 53 | 57 | 64 | 40 | 47 | 51 | 60 | 64 | 34 | 48 | 55 | 61 | 94 |
| 16 |  |  |  |  |  | 36 | 44 | 53 | 56 | 86 | 38 | 46 | 58 | 70 | 112 | 51 | 68 | 69 | 70 | 75 |
|  | | | | | | | | | | | | | | | | | | | | |
| ***EFSA AI*** | 25 | | | | | 25 | | | | | 80 | | | | | 80 | | | | |
| ***US AI*** | 30 | | | | | 30 | | | | | 75 | | | | | 75 | | | | |
| ***Prevalence inadequate intake^1^*** | low | | | | | low | | | | | no statement | | | | | no statement | | | | |

^1^Prevalence of inadequate intake: low = when the median intake is above the AI the prevalence of inadequate intake is likely to be low; no statement = when the median intake is below the AI the adequacy cannot be evaluated
